# Transfer of Learning from Vision to Touch: A Hybrid Deep Convolutional Neural Network for Visuo-Tactile 3D Object Recognition

**DOI:** 10.3390/s21010113

**Published:** 2020-12-27

**Authors:** Ghazal Rouhafzay, Ana-Maria Cretu, Pierre Payeur

**Affiliations:** 1 School of Electrical Engineering and Computer Science, University of Ottawa, Ottawa, ON K1N 6N5, Canada; ppayeur@uottawa.ca; 2 Department of Computer Science and Engineering, Université du Québec en Outaouais, Gatineau, QC J8X 3X7, Canada; Ana-Maria.Cretu@uqo.ca

**Keywords:** 3D object recognition, transfer learning, machine intelligence, convolutional neural networks, tactile sensors, force-sensing resistor, Barrett Hand

## Abstract

Transfer of learning or leveraging a pre-trained network and fine-tuning it to perform new tasks has been successfully applied in a variety of machine intelligence fields, including computer vision, natural language processing and audio/speech recognition. Drawing inspiration from neuroscience research that suggests that both visual and tactile stimuli rouse similar neural networks in the human brain, in this work, we explore the idea of transferring learning from vision to touch in the context of 3D object recognition. In particular, deep convolutional neural networks (CNN) pre-trained on visual images are adapted and evaluated for the classification of tactile data sets. To do so, we ran experiments with five different pre-trained CNN architectures and on five different datasets acquired with different technologies of tactile sensors including BathTip, Gelsight, force-sensing resistor (FSR) array, a high-resolution virtual FSR sensor, and tactile sensors on the Barrett robotic hand. The results obtained confirm the transferability of learning from vision to touch to interpret 3D models. Due to its higher resolution, tactile data from optical tactile sensors was demonstrated to achieve higher classification rates based on visual features compared to other technologies relying on pressure measurements. Further analysis of the weight updates in the convolutional layer is performed to measure the similarity between visual and tactile features for each technology of tactile sensing. Comparing the weight updates in different convolutional layers suggests that by updating a few convolutional layers of a pre-trained CNN on visual data, it can be efficiently used to classify tactile data. Accordingly, we propose a hybrid architecture performing both visual and tactile 3D object recognition with a MobileNetV2 backbone. MobileNetV2 is chosen due to its smaller size and thus its capability to be implemented on mobile devices, such that the network can classify both visual and tactile data. An accuracy of 100% for visual and 77.63% for tactile data are achieved by the proposed architecture.

## 1. Introduction

Literature from neuroscience confirms that visual and haptic object recognition rely on similar processes in terms of categorization, recognition and representation [1]. Many researchers suggest the possibility that a shared neural circuitry in the human brain is trained to do both [2,3,4]. The cortical areas in the ventral and dorsal streams of the brain are consistently activated for visual as well as haptic data processing [5]. Moreover, in many cases, humans are able to haptically recognize objects for which they have learned their characteristics by only using vision. As such, this paper aims to test these assumptions in a realistic scenario for a robotic haptic 3D object recognition task.

Haptic perception differs from tactile perception in the sense that it refers to both kinaesthetic data acquired from joints and muscles, as well as tactile data sensed by mechanoreceptors in human skin, including pressure, torsion, vibration, roughness and force. Most of the robotic arms available in laboratories for haptic object manipulation are equipped with a variety of sensors supplying different sorts of information considered as haptic data. Robot joint angles, finger angles, temperature signals, pressure signals [6,7] and tactile images obtained as 2D arrays [8,9,10] presenting the texture and local characteristics of object surface are some examples. According to neuroscience research, haptic object processing itself relies on at least two different neural pathways, one interpreting local geometrical properties and the other the material properties of objects such as hardness, roughness, compliance and temperature [5]. The first neural pathway is possibly a point of convergence between vision and touch [5]. In this work, we only consider tactile data capturing local geometrical properties of objects and, in compliance with other works in the literature, refer to them as tactile images. Tactile images usually require a prior feature extraction step before further processing and classification. Despite the success of traditional feature extraction techniques such as 2D wavelet transform [11] and contourlet transform [12,13] for feature extraction from tactile images, training a deep neural network architecture to learn to extract features from tactile images remains a topic of interest in the deep learning era [13,14].

The fast advancement of deep learning-based computational architectures in recent years has made these architectures a promising solution in many robotic and computer vision tasks. Convolutional neural networks (CNN) are widely accepted as the artificial counterpart of human vision for a variety of robotic applications. However, training a deep CNN on tactile data from scratch is not easy due to the fact that deep learning requires large datasets of sample data to develop an efficient model, while available tactile datasets are of small size compared to image databases, due to the fact that tactile data acquisition tends to be a difficult and time-consuming task. This motivates us to study the possibility of transferring learning from visual data to tactile data using a pre-trained deep CNN on visual data in order to recognize 3D objects using tactile data. On the other hand, a collection of tactile sensors with different technology and working principles are nowadays available on the market, each with its specific resolution and data architecture [15]. The potential for the transfer of learning from visual data for each of these technologies is questionable, thus motivating our interest in this research topic.

In this work, we take advantage of five different pre-trained deep CNN architectures, including Alexnet, GoogLeNet, VGG16, Resnet50 and MobileNetV2. In a first experiment, we finely tune the weights in all layers on tactile data in order to recognize 3D objects based on these data. In a second experiment, we freeze the weights for all layers (i.e., we set the learning rate for those layers to zero) and only finely tune the last three layers, such that identical visual filters are applied to extract features from tactile data for the purpose of classification. The framework is tested on four different technologies for tactile data acquisition, including data coming from a GelSight sensor [16], a force-sensing resistor (FSR) tactile sensor array [10], a BathTip tactile sensor [9] and from the tactile sensing electrodes distributed across fingers available in the Barrett Hand [17]. The main contributions of the work in this paper are the following: (1) Demonstrate how transferable visual features are to tactile features for four different technologies of tactile sensors; (2) Measure the similarity between visual and tactile features; (3) Determine which convolutional layers in MobileNetV2 are most altered in order to allow for the transfer of the model to touch; and based on that, (4) Propose a novel hybrid architecture to perform both visual and tactile 3D object recognition.

The paper is structured as follows: Section 2 discusses the relevant works from the literature. Further details about the adopted datasets and CNN architectures are provided in Section 3 and Section 4, respectively. Section 5 reports on the experimental setups. Results are monitored and discussed in Section 6. Section 7 proposes a hybrid architecture to classify both visual and tactile data. Section 8 concludes the work.

## 2. Related Works

The literature on haptic object recognition comprises work related to haptic data acquisition techniques as well as data processing and classification approaches. Inspired by visuo-haptic integration in the human brain, many researchers devoted their research efforts to the enhancement of robot sensing solutions by combining visual and tactile data. It has now been demonstrated that direct integration of haptic and visual data can increase object recognition rate [14,18]. Other research work suggests that visual data can contribute as a data selection strategy to guide the process of haptic data acquisition [13,19].

Neural networks, especially in their deep version, are on a continuous pathway of research progress and have found their application in both robot vision and touch technologies. Luo et al. [8] propose a hybrid architecture based on deep convolutional neural networks learning features from visual and tactile data separately. Maximum covariance analysis is then employed to achieve a joint latent space. These features are finally used for multiclass classification. Lee et al. [20] take advantage of generative adversarial networks [21] to produce visual counterparts of tactile images captured by a GelSight sensor. Comparing the generated visual data and real visual data confirms the reliability of the generated data. In our previous research [13], we trained a 3D CNN on sequences of tactile data captured by moving a tactile sensor around an object to classify the corresponding object based on them. Gandarias et al. [22] train a similar architecture on sequences of tactile data captured from deformable objects when subjected to different pressures to successfully classify nine objects. Zheng et al. [23] train a fully convolutional neural network to classify different materials using haptic textures and acceleration signals acquired by moving a probe over the materials.

A number of recent research works are studying the transfer of learning from pre-trained CNN architectures on images to tactile data. Alameh et al. [24] use seven different pre-trained CNN architectures and finely tune the fully connected layer to classify 400 by 400 tactile images generated using tactile data from a 16 by 16 piezoelectric sensor. Gandarias et al. [25] adopt CNN architectures for feature extraction from a database of large-scale, high-resolution tactile images captured with a piezoresistive array. They compare the results where a fully connected layer is employed to classify extracted features by convolutional layers in a Resnet50 with the case where a support vector machine follows features extracted using a VGG16 architecture. Resnet50 results in slightly higher performance. Moreover, they customize three other CNN architectures, two with a different number of convolutional layers and one by adding residual feedback and train them on tactile data only. They conclude that the classification accuracy is essentially a function of tactile image spatial resolution by running experiments on downsampled versions of tactile images. In addition to the tactile features of their dataset at the texture level, the large-scale of their tactile data leads to capturing the general shape of objects, which is a leading characteristic for both visual and tactile object recognition.

Taking inspiration from neuroscience, which suggests a similarity between visual and tactile features for form [26], shape processing [4] and possibly surface roughness [26], in this work, by comparing data acquired from different technologies of tactile sensors, we aim to reveal how compliant the extracted features from different tactile images (at the texture level only) are with visual features and, as a consequence, how transferrable visual features are to tactile data. Tactile images used in this work do not provide any information about object shapes or their global form, which are the key characteristics in the object recognition task. Further experiments are carried out to see how a hybrid network can be developed to recognize 3D objects from both visual and tactile data. For this purpose, we measure how weight values in convolutional layers of a CNN are updated while fine-tuning the network on tactile data. A hybrid network is then proposed by introducing additional convolutional layers for tactile data. The framework is tested on a dataset of 3D models to recognize objects from visual and tactile data, where visual data are collected using a Matlab virtual camera and tactile data are simulated by a sensor introduced in [26]. Since visual data are not available for the other datasets studied in this paper, the hybrid network is only developed and tested for the dataset of 3D object models (Section 3.3).

It is worth mentioning that previous works from the literature either train a CNN from scratch or by transfer learning on tactile images to recognize objects only from touch, while the main contribution of the current research is to study the links between vision and touch in an attempt to converge the visual and tactile processing units in a robot.

## 3. Datasets and Data Processing

In this section, we present the four datasets that we have used to explore the possibility of transfer learning from vision to touch and their underlying technology of tactile sensors.

### 3.1. ViTac Dataset

ViTac [8] is a dataset of visual and tactile data obtained from 100 pieces of clothes with different materials and textures. A GelSight sensor is used to acquire tactile data, and visual data are captured by a camera with its image plane perpendicular to the clothing material. GelSight sensor is an optical tactile sensor using a piece of elastomeric gel with a reflective membrane coat on top, which enables it to capture fine geometrical textures as a deformation in the gel. A series of LEDs with RGB color illuminates the gel such that a camera can record the deformation. In this study, we run experiments on 12 classes of tactile data from the dataset, which is publicly available in [27].

### 3.2. VT-60 Dataset

VT-60 dataset [9] is an open-access dataset of tactile data captured using the BathTip [9] tactile sensor. The BathTip sensor is another optical tactile sensor consisting of an elastic silicone hemispherical membrane mounted at the end of the encasing of a digital camera. Any deformation in the membrane in contact with objects is captured using the camera. In comparison with the GelSight sensor, this sensor is less sensitive to fine tactile features. The dataset includes data from 10 classes, namely, stapler, empty bottle, ball, soft toy, shoe, box, mug, full bottle, bowl and can.

### 3.3. FSR Tactile Array and High-Resolution Simulated FSR Sensor

**Figure 1:**
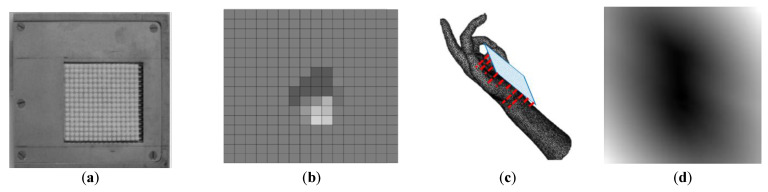
(**a**) Force-sensing resistor array, (**b**) example of tactile data, (**c**) simulated tactile sensor for the virtual environment, and (**d**) an example of a simulated tactile image.

The FSR tactile sensor consists of a 16 by 16 array of force-sensing resistors, covered by a protective elastic array and placed on an area of 6.5 cm^2^. In direct contact with an object, the geometric profile captured by the elastic overlay of the sensor is first mapped into force components through a profile-to-force transductor, and then the applied forces are mapped into electrical signals to form a tactile image [10]. The FSR tactile sensor, as well as an example of the acquired tactile image, are illustrated in Figure 1a,b. A virtual counterpart of this sensor was also developed to simulate the acquisition of tactile data from 3D object models [28] and allow to study the impact of tactile imprints’ quality and size as well as to plan the real acquisition of data. The virtual sensor allows modifying both the number of sensing elements and the distance between them. As such, it can be used to establish experimental setups and proof of concept for various tasks prior to running experiments with real sensors, which is, in general, long and tedious due to the need to move the robotic arm carrying the tactile sensor and bring the sensor in contact with the object at multiple locations. In order to simulate tactile images, as depicted in Figure 1c, we modeled the surface of the sensor as a tangential plane to the object surface at the probing location, shown in blue in the figure. This is justified by the fact that the quality of tactile imprints is better when the imprint is captured in the direction of the local normal on the surface of the object. Distances between the virtual object surface and sensing elements on the plane are computed and normalized in the range of 0 to 1 to generate a tactile image similar to the example in Figure 1d. Additional details about this virtual sensor are provided in [28]. Tactile imprints of a resolution of 128 by 128 are simulated for experiments in this paper. The dataset from the simulated FSR sensor is collected from a set of 30 virtual objects in the form of triangular meshes belonging to 6 classes, namely, bird, chair, hand, head, plane and quadruped. An equal number of tactile images is acquired from each object in the dataset, and a split of 75/25 is used for training and testing.

### 3.4. BiGS Dataset

The BiGS dataset [6] is a dataset of tactile data captured by a Barrett Hand while grasping three different objects, namely a box, a ball and a cylinder. The robot hand has three fingers, each equipped with impedance sensing electrodes. Electrode values are sampled at 100 Hz, and these values can be interpreted and mapped to produce a tactile image of size 7 by 3.

**Figure 2:**
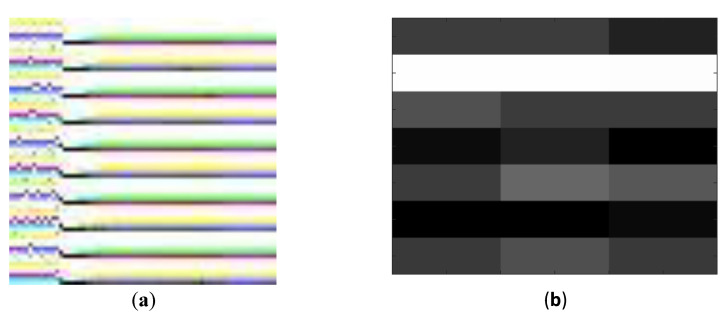
(**a**) An example of 70 by 70 generated RGB tactile image from the BiGS (BioTac Grasp Stability) dataset, and (**b**) an example of 7 by 3 instantaneous electrode reading.

The BiGS dataset contains both success and failure in grasping cases. In the context of this work, we only consider success cases since we use the data for the purpose of 3D object recognition and success cases give data of better quality for this purpose. Initial experimentation with the dataset demonstrated that deep CNNs trained on instantaneous 7 by 3 tactile images acquired while grasping failed to recognize the objects. We believe that this is due to the low-resolution of tactile data. As such, in this work, in order to produce higher resolution tactile images for the input of deep CNNs, we use the first 700 sampled values of each electrode while grasping an object and reshape the resulting 7 by 3 by 700 array into a 70 by 70 RGB image (70 by 70 by 3). The appropriateness of using such an approach for reshaping temporal data is confirmed in the literature for tactile data [24]. An example of such a tactile image is illustrated in Figure 2a. Figure 2b illustrates an example of an instantaneous (7 by 3) electrode reading from the Barrett Hand. Results reported in Section 6 are all obtained using the 3 channel, 70 by 70 images.

## 4. Transfer of Learning Using CNNs

Popular deep CNN architectures are constructed by a series of convolutional layers following an image input layer. Several max-pooling layers can be introduced in between the convolutional layers, with the purpose of both reducing the feature map size and improving the translational invariance property of the network. Fully connected layers at the end of the network are trained to map extracted features from inputs into classification outputs. When training a CNN, weights and bias values in convolutional layers are adjusted to extract relevant features from the dataset. Transfer learning is a frequently used technique in deep learning, in which a pre-trained network is used as a starting point for readaptation of the network to other tasks. It allows for the rapid progress of the training process on new datasets, and it can improve the performance of the target network. Transfer learning will only work if the features are general and suitable for both the base task and target task [29]. In this work, inspired by neuroscience and in pursuit of recent works on transferring learning from vision to touch [24,25], we study the transferability of visual features to tactile features for different setups, different tactile sensor technologies and different deep CNN architectures in order to subsequently recognize the 3D objects based on tactile features. AlexNet [30], GoogLeNet [31], VGG 16 [32], Resnet50 [33] and MobileNetV2 [34] are the five pre-trained CNN architectures that we chose for experimentation in this work. AlexNet, proposed in 2012 and one of the precursors of deep CNNs for transfer learning, consists of 25 layers, including 5 convolutional layers. Since then, a variety of deep architectures were proposed to enhance its performance. GoogLeNet allows applying convolutional masks of different sizes together with a max-pooling operation in a single layer as an inception module. VGG16 adds up more layers and thus consists of 13 convolutional and 3 fully connected layers. ResNet50 and its deeper versions facilitate backpropagation of the gradient in CNNs and thus improve the performance of very deep architectures by introducing the concept of residual feedbacks. MobileNetV2 offers comparable performance with other deep CNNs, but has a small size and can be implemented even on mobile devices; hence it is much more suitable for robotic tasks.

## 5. Experimental Setup

For each dataset and each architecture, two networks are trained. The first network replaces the last three layers (i.e., fully connected layer, followed by a SoftMax and classification layers) of each architecture, its role being to adapt the network for specific classification tasks according to the number of classes in each dataset. It uses the pre-trained CNNs on ImageNet [35], a large and popular visual database frequently used for transfer learning, as a start point and finely tunes the network weights in all layers on each tactile dataset. The other network freezes the weight values of all CNN layers and only finely tunes the fully connected layers for the classification of tactile data such that the same convolutional filters employed to extract visual features are applied to extract tactile features. It is important to clarify that both AlexNet and VGG 16 consist of three fully connected layers, while the other networks studied in this work have a single, fully connected layer. As such, the exact convolutional filters trained on ImageNet are applied to tactile datasets. In each case, several networks are trained by finely tuning the learning rate (LR) to achieve the best performance. A 75–25% split of data is used for training and testing. All grayscale tactile images are transformed into three-channel images by assigning the gray values to the red channel and zero-padding the green and blue channels. Our prior experimentation into assigning grayscale images to all of the three RGB channels as well as to each of the three channels separately while setting the other channels to zero demonstrated no significant influence on the classification accuracy, so in the remaining of the paper, we padded the green and blue channels with zeros. To prevent the networks from overfitting, training data are augmented by random reflection, translation and scaling of the dataset using the ImageDataAugmenter tool of Matlab. Training data are shuffled at every epoch. A stochastic gradient descent with a momentum of 0.9 is used for training. All networks are trained using MATLAB R2019a platform and on a single Nvidia GeForce RTX 2070 GPU card and using a large enough number of epochs such that no further improvement can be seen in the learning curve. Since the number of object classes in the studied datasets is not consistent, all the obtained classification accuracies (ACC) are also reported with respect to a random guess ($\frac{1}{\mathrm{number}\;\mathrm{of}\;\mathrm{objects}}\times 100$), where the random guess is 10% for ViTac, 8.33% for VT60, 16.67% for FSR array, and 33.3% for the BiGS dataset, respectively.

## 6. Classification Results and Discussion

Table 1, Table 2, Table 3, Table 4 and Table 5 report the performance of each network for the 5 studied databases in terms of accuracy. For all types of tactile data, the network can learn the corresponding tactile features at a certain level by finely tuning the convolutional layer weights. Similar to other applications of deep learning, Resnet50 outperforms other architectures in all cases, which is due to the deeper architecture of Resnet 50 as well as the presence of residual connections facilitating the training process. MobileNetV2, with considerably smaller model size, is demonstrated to offer a comparable performance to Resnet50 when the network weights are finely tuned for tactile data. Similar to Resnet 50, MobileNetV2 takes advantage of residual connections. The accuracy differences between MobileNetV2 and Resnet50 vary between 0.59% and 3.33% for the finely tuned weights networks.

In the case of optical tactile sensors, i.e., BathTip (Table 1, column 4, ACC above random guess) and GelSight (Table 2, column 4), features are more transferable from vision to touch. This is demonstrated by the fact that CNNs trained on data from optical tactile sensors succeed in achieving an accuracy of up to 82.88% and 90.64%, respectively, above a random guess. The accuracy above a random guess is lower for other technologies, i.e., 28.09% for an FSR array (Table 3, column 4), 65.23% for the simulated FSR of 128 by 128 tactile image resolution (Table 4, column 4) and 58.76% for the Barrett Hand (Table 5, column 4). Even in the cases where the visual filters (convolutional layers with frozen weights) are directly applied to optical tactile data (Table 1 and Table 2, column 7), all networks succeed to classify tactile data with a considerable margin above random guess, i.e., up to 55.34% for the BathTip sensor (VT-60 dataset), and 84.1% for the GelSight sensor (ViTAC dataset). This confirms the idea that vision and touch share highly similar features at the fine texture level. Among the studied technologies of tactile sensors in this work, only optical tactile sensors are capable of capturing fine texture level features. For the FSR sensor array, the accuracy value remains around a random guess with frozen weights (Table 3, column 7), and only for simulated tactile data where the resolution and precision of the sensor are increased; tactile data can become more distinguishable according to visual features (Table 4, column 7). We believe the shortcoming of such tactile sensors is mainly due to their low-resolution, which is also confirmed by the Barrett Hand dataset in its initial form, i.e., 7 by 3 instantaneously electrode readings (Section 3.4).

Our prior experiments demonstrated that tactile images captured as instantaneous values of impedance electrodes on Barrett Hand fingers are not classifiable using transfer learning. All accuracies remained around random guess when a single image of size 7 by 3, as the one shown in Figure 2b, was used for classification. This can be both due to the low-resolution of tactile images and the high similarity between the tactile properties of the three objects contained in this dataset. When working with robotic arms, kinesthetic cues such as finger angles to grasp the object tend to be more informative about the global shape of objects. We succeeded in training CNNs on tactile images generated from sequences of electrode values, as explained in Section 3.4. Results are reported in Table 5. Maximum accuracy of 58.76% above a random guess is achieved for finely tuned CNNs on tactile data and 49.99% with frozen weight CNNs.

**Figure 3:**
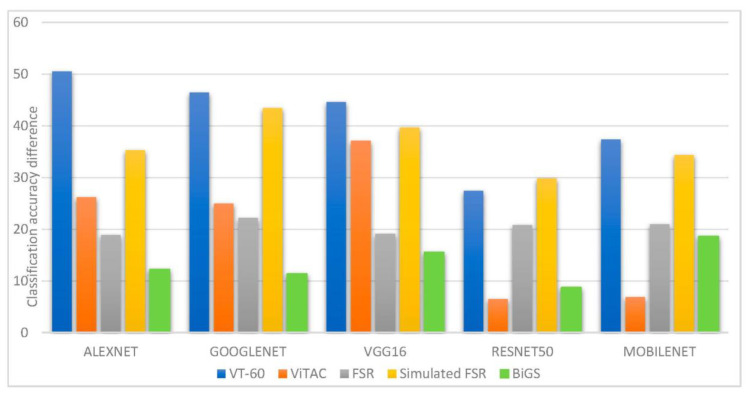
Accuracy differences between convolutional neural networks (CNNs) with frozen weights and CNNs with fine-tuned weights.

To better interpret the influence of tuning networks on classification accuracies, Figure 3 visualizes the accuracy differences between networks with finely tuned weights and frozen weights for each dataset.

**Figure 4:**
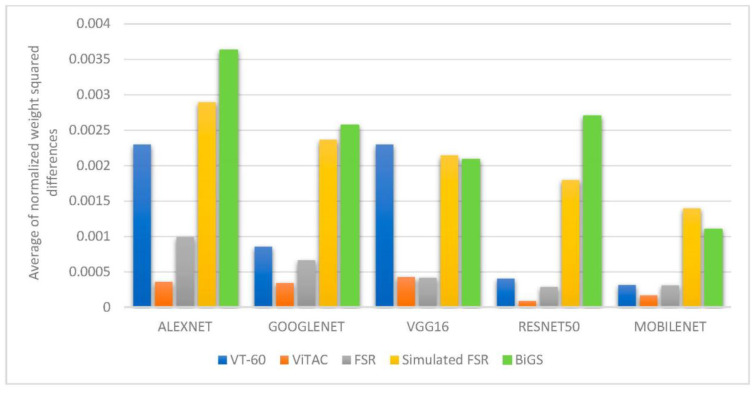
Average normalized weight differences between CNNs with frozen weights and CNNs with fine-tuned weights.

To further analyze the feature extraction process in different networks, we also measure how much weight values in convolutional layers are modified from the base networks (trained on ImageNet) to extract features from tactile data. For this purpose, for each CNN, we first normalize the weight values of each convolutional layer to values between 0 and 1, and then we measure the weight differences between each convolutional layer of the network with frozen weights and the corresponding convolutional layer in the fine-tuned network. The average normalized weight squared differences are computed for each network and each dataset. Results are reported in Figure 4. In this section, Figure 3 and Figure 4 are jointly interpreted to draw conclusions from experiments.

One can notice that in the BiGs dataset, shown in green in Figure 3 and Figure 4, in spite of the large weight updates required to adapt the network for tactile data classification (Figure 4), the progress in accuracy rates is relatively low (Figure 3), which means that the loss function cannot be efficiently minimized. For the two tactile datasets using optical sensors, shown in blue and orange in Figure 3 and Figure 4, the weight updates are relatively low (except for Alexnet and VGG16 on the VT-60 dataset), confirming that the difference between convolutional filters to extract visual and tactile features is low, and thus suggesting that extracted features are similar.

It is important to note that, in spite of having the best performance, Resnet50 shows in most cases the smallest updates both in weights and accuracy values. The reason can be found in the deep architecture of Resnet50 that allocates smaller weight updates to each layer, and thus, the average normalized weight differences are lower.

In deep CNN architectures, convolutional layers with larger kernels are usually placed in earlier layers to extract general features from data, such as color and edges, and are not particular to a specific dataset [28]. Features from the later layers, closer to the output, are of higher level and are mostly updated to adapt the network to achieve a specific task.

Relying on the MobileNetV2 architecture, which can be implemented on mobile devices, we studied which convolutional layers in MobileNetV2 are mostly updated to transfer learning from vision to touch. For this purpose, we measured the average difference between corresponding convolutional layer weights for each convolution or grouped convolution layer. Measuring the weight updates in MobileNetV2 suggests that convolutional layers at earlier layers are more altered while tuning the network weights on tactile data. Knowing which convolutional layers are mostly altered to adapt a pre-trained CNN for classification of tactile data can be useful to generate a hybrid CNN architecture handling both visual and tactile sensing in such a way that further layers can be added in parallel to a base network with fine-tuned weights to perform tactile object recognition. Such an architecture is developed and tested in Section 7.

## 7. Hybrid Deep Architecture for Object Recognition

Comparison of weight updates in MobileNetV2 on tactile data suggests that the early layers play a decisive role in the performance of the network and need to be tuned on tactile data. To make sure that the layers we are freezing in order to develop the hybrid neural network will lead to the best possible performance, a number of MobileNetV2 networks are trained and tested with a different heuristic combination of frozen layers (both early layers and final layers of the network). Obtained classification accuracies are reported in Table 6.

One can notice that freezing the layers 18 to 139 of the network while fine-tuning weights of the remaining layers results in the closest possible accuracy to the case where the weights of all layers in the network are tuned on the tactile data. MobileNetV2 consists of sixteen so-called “convolutional building blocks” [34]. Layer 17 is the last layer of the first convolutional block from which a residual connection directly adds the output of this layer with the output of the second residual block. Our empirical experiments suggest that only the first and the last convolutional building blocks need to be tuned on tactile data to achieve the maximum accuracy in classification. A similar setup on visual data gives a 100% accuracy, which is equal to the case where the weights are tuned on visual data. These results also confirm the experiments from the previous section, suggesting that tuning of the weights of the early layers, i.e., layers 1 to 17, of the network is pivotal for transfer learning to tactile data.

**Figure 5:**
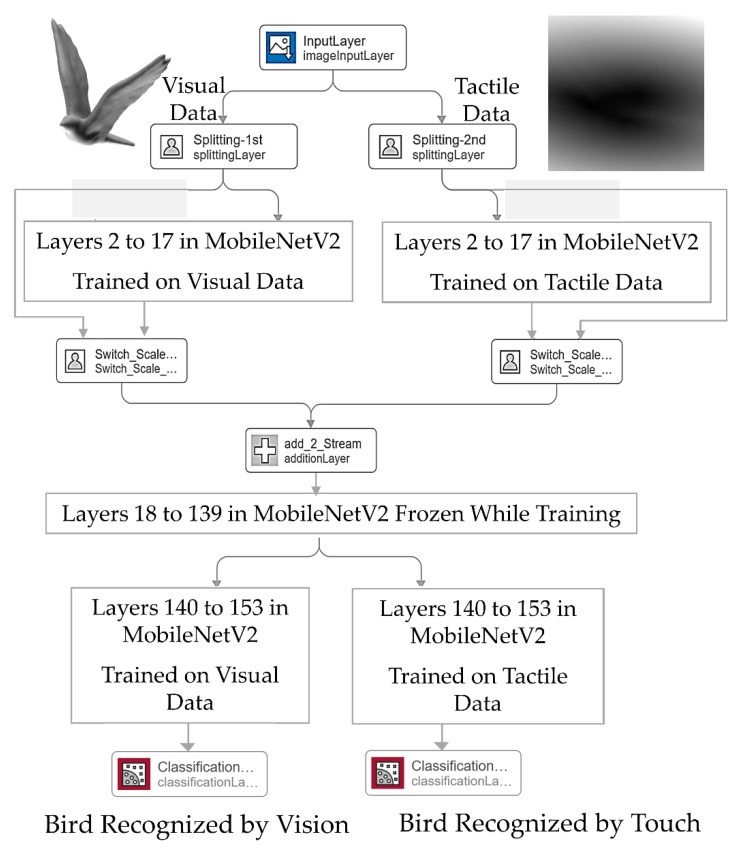
Architecture of the proposed hybrid visuotactile object recognition stage.

Accordingly, the hybrid network illustrated in Figure 5 is developed by stitching different layers of two MobileNetV2 architectures trained on visual and tactile data with layers 18 to 139 frozen. This hybrid architecture is developed in Matlab R2020a platform using the Deep Network Designer. It is worth mentioning that in this paper, we address all layers according to their labeling in Matlab Deep Network Designer.

**Figure 6:**
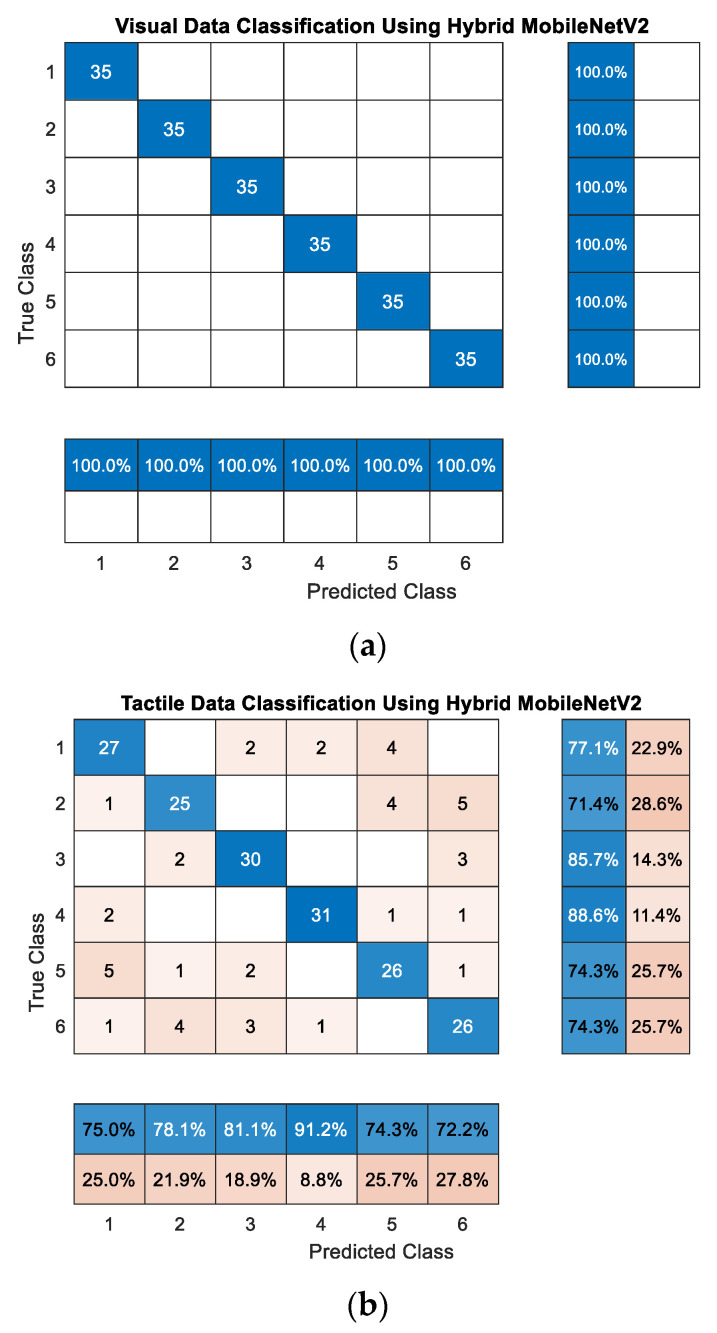
Confusion matrices for visuotactile hybrid object recognition architecture for (**a**) visual data and (**b**) tactile data.

In order to produce a network with two input streams, we set an image input layer of size 224 by 224 by 6 followed by two custom-designed splitting layers separating the two input streams, i.e., visual and tactile inputs. The initial input to the network is in the form of a 6-channel image of size 224 by 224. The first three channels are allocated to RGB values of visual data, and the last three channels contain tactile data. If a visual image is to be classified, the tactile channels are set to zero and vice versa. The image input layer normalizes the input data using z-score, where the mean and standard deviation of each data channel is extracted and set from the initially trained networks. The input data passes through layers 2 to 17 of the associated MobileNetV2 layers trained on visual/tactile data. Two custom switch-scale layers, as illustrated in Figure 5, are introduced to eliminate the output of layers 2 to 17 for the stream that is not supposed to participate in classification (i.e., the stream with the input from zero-channels). The switch-scale layer takes two inputs. Input 1 is the output of the splitter layer, and input 2 takes the activations from Layer 17 of the MobileNetV2. The variance across each of the three-channel data from input 1 is measured to determine if the data for that stream corresponds to the zero channels. If the variances for all three channels are equal to zero, which means the data are from zero-channels, a zero array of the size of the activations at layer 17, i.e., 56 by 56 by 24, is generated and multiplied with the activations from layer 17 (i.e., input 2 of the switch-scale Layer). Otherwise, an array of ones of size 56 by 56 by 24 is used to keep the activations from layer 17 unchanged. The output of the two streams is then summated and passed through layers 18 to 139 with frozen weights. From layer 140 to the final layer, i.e., layer 154, as illustrated in Figure 5, the information passes through two parallel paths with finely tuned weights on the corresponding data for classification purposes. It is worth mentioning that the stitched network is only used for prediction purposes, and the weights remain unchanged. The hybrid network outputs an accuracy of 100% on visual object recognition and an accuracy of 77.63% on tactile data while achieving a compression ratio of 1.4284 (i.e., two separate networks occupy the memory of 16,644 KB while the hybrid network has a size of 11,652 KB). It is worth mentioning that this performance is comparable with the case where all layers of MobileNetV2 are tuned on tactile data (i.e., 78.57%) and is 34.76% higher than the case where all layers of the network are frozen. Figure 6a,b depicts the confusion matrix of the hybrid visuotactile object recognition architecture on visual and tactile data where the objects 1 to 6 correspond to classes: bird, chair, hand, head, plane and quadruped, respectively from the dataset described in Section 3.3.

## 8. Conclusions and Future Work

In this paper, we studied the possibility of transferring learning from vision to touch using deep CNN architectures, validating the idea that visual and tactile data share similar features at certain levels. The idea is tested on different types of tactile sensors capturing local geometry of objects and using five state-of-the-art pre-trained CNNs. Optical tactile sensors, due to their higher resolution, respond better to transfer of learning from vision, and they succeed in achieving accuracy up to 90.64% above random guess. We believe that the resolution of tactile data has a pivotal role in tactile object recognition. Increasing the resolution of a 16 by 16 FSR array to 128 by 128 by simulation results in average growth of 35.60% in classification accuracy. Similarly, preprocessing tactile images from a Barrett Hand to generate 70 by 70 images yields a 92.06% accuracy for the classification of three objects. Transfer learning from vision to touch can help to merge visual and tactile sensory circuitry in autonomous robots and to develop a dual learning strategy to train them for both visual and tactile understanding. Further analysis is carried out to identify which convolutional layers are more altered in MobileNetV2 architecture to adapt the network for tactile data classification. Our experiments suggest that the convolutional weights in the first and the last convolutional building blocks in the architecture of MobileNetv2 have a decisive role in classification accuracy. Based on this investigation, a hybrid CNN architecture with visual and tactile input streams is developed with fine-tuned weights for each task at the first and the last convolutional building blocks and domain invariant features at intermediate layers of the network to classify both visual and tactile data from a dataset of 3D models. Extra layers are introduced to control the flow of information for each source of sensory input. Accuracies of 100% and 77.63% are achieved, respectively, for object recognition by vision and touch. The accuracy value achieved for tactile data with the proposed architecture is 34.76% above the case where a pre-trained MobileNetV2 on visual data with frozen weights is used for object recognition by touch.

These findings can contribute to assimilating the sensory perception in robots to human cognition by converging visual and tactile processing units. Future work of the current study will consist of improving the current hybrid visual and tactile model with additional components of tactile sensing such as high-frequency vibration and temperature and thus expanding the hybrid network for more data processing tasks.

## Figures and Tables

**Table 1:** Classification results for VT-60 dataset. All networks are trained on a minimum batch of size 16 and for 20 epochs.

	Fine-Tuned Weightson Tactile Data			Frozen Weight Networks		
CNN	LR	ACC	ACC above Random Guess	LR	ACC	ACC above Random Guess
AlexNet	1 × 10^−4^	82.99%	72.99%	1 × 10^−5^	32.43%	22.43%
GoogLeNet	5 × 10^−4^	87.56%	77.56%	1 × 10^−5^	41.10%	31.10%
VGG16	1 × 10^−4^	85.70%	75.70%	1 × 10^−5^	41.04%	31.04%
ResNet50	1 × 10^−4^	92.29%	82.29%	1 × 10^−4^	65.34%	**55.34%**
MobileNetV2	1 × 10^−4^	92.88%	**82.88%**	5 × 10^−5^	55.45%	45.45%

**Table 2:** Classification results for ViTac dataset. All networks are trained on a minimum batch of size 16 and for 10 epochs.

	Fine-Tuned Weightson Tactile Data			Frozen Weight Networks		
CNN	LR	ACC	ACC above Random Guess	LR	ACC	ACC above Random Guess
AlexNet	1 × 10^−4^	96.77%	88.44%	1 × 10^−4^	70.50%	62.17%
GoogLeNet	0.001	97.25%	88.92%	1 × 10^−4^	72.21%	63.88%
VGG16	1 × 10^−4^	94.09%	85.76%	1 × 10^−4^	56.95%	48.62%
ResNet50	1 × 10^−4^	98.97%	**90.64%**	1 × 10^−4^	92.43%	**84.1%**
MobileNetV2	1 × 10^−4^	96.77%	88.44%	1 × 10^−4^	89.27%	80.94%

**Table 3:** Classification results for tactile data collected by 16 by 16 force-sensing resistor (FSR) array. All networks are trained on a minimum batch of size 16 and for 10 epochs.

	Fine-Tuned Weightson Tactile Data			Frozen Weight Networks		
CNN	LR	ACC	ACC above Random Guess	LR	ACC	ACC above Random Guess
AlexNet	1 × 10^−5^	36.19%	19.52%	1 × 10^−5^	17.14%	0.47%
GoogLeNet	1 × 10^−5^	39.57%	22.9%	1 × 10^−5^	17.62%	0.95%
VGG16	1 × 10^−5^	38.57%	21.9%	1 × 10^−5^	19.52%	2.96%
ResNet50	1 × 10^−5^	44.76%	**28.09%**	1 × 10^−5^	24.29%	**7.62%**
MobileNetV2	1 × 10^−5^	41.43%	24.76%	1 × 10^−5^	20.48%	3.81%

**Table 4:** Classification results for simulated 128 by 128 FSR tactile data. All networks are trained on a minimum batch of size 16 and for 10 epochs.

	Fine-Tuned Weightson Tactile Data			Frozen Weight Networks		
CNN	LR	ACC	ACC above Random Guess	LR	ACC	ACC above Random Guess
AlexNet	2 × 10^−4^	63.81%	47.14%	1 × 10^−4^	28.57%	12.2%
GoogLeNet	0.005	79.52%	62.85%	1 × 10^−4^	35.24%	18.57%
VGG16	5 × 10^−4^	74.76%	58.09%	1 × 10^−4^	34.76%	18.09%
ResNet50	1 × 10^−4^	81.9%	**65.23%**	1 × 10^−4^	**52.38%**	35.71%
MobileNetV2	5 × 10^−4^	78.57%	61.9%	1 × 10^−4^	43.81%	27.14%

**Table 5:** Classification results for BiGS data set. All networks are trained on a minimum batch of size 16 and for 10 epochs.

	Fine-Tuned Weightson Tactile Data			Frozen Weight Networks		
CNN	LR	ACC	ACC above Random Guess	LR	ACC	ACC above Random Guess
AlexNet	1 × 10^−4^	85.24%	51.94%	1 × 10^−5^	72.98%	39.68%
GoogLeNet	0.001	83.57%	50.27%	1 × 10^−5^	72.14%	38.84%
VGG16	1 × 10^−4^	89.69%	56.39%	1 × 10^−4^	74.09%	40.79%
ResNet50	0.001	92.06%	**58.76%**	0.001	**83.29%**	49.99%
MobileNetV2	0.001	90.67%	57.37%	1 × 10^−4^	72.01%	38.71%

**Table 6:** Classification accuracy of MobileNetV2 with an overall of 154 layers on tactile data for different frozen layers.

Classification Accuracy	Frozen Layers
43.81%	All layers frozen
62.86%	1:150
64.29%	1:144
68.10%	1:134
65.71%	10:150
74.29%	10:144
68.57%	16:138
**77.63%**	**18 to 139**
78.57%	No frozen layer

## Data Availability

Data sharing is not applicable to this article.
